# Engaging traditional, complementary and alternative health providers for essential health service delivery in India: a policy analysis

**DOI:** 10.1186/1753-6561-6-S5-O31

**Published:** 2012-09-28

**Authors:** Lakshmi K Josyula, Kabir Sheikh, TN Sathyanarayana, Devaki Nambiar, Venkatesh Narayan, John Porter

**Affiliations:** 1 Indian Institute of Public Health, Hyderabad, India; 2 Public Health Foundation of India, New Delhi, India; 3 London School of Hygiene and Tropical Medicine, UK

## Introduction

Efforts to engage traditional, complementary, and alternative (TCA) health providers in the public health workforce are gaining increasing attention in India. Evidence from studies evaluating the efforts to integrate TCA health providers into the formal public health system highlights numerous lacunae in policy and practice. This study was undertaken to diagnose operational and ethical challenges in the implementation of current policies for integration of TCA health providers in order to derive lessons to strengthen integration strategies.

## Methods

We used qualitative data collection methods and an interpretive policy analysis approach in three states of India: Kerala, Delhi, and Meghalaya. Health policy documents, including bills, acts, orders, inter-institution communications, and publicly available material on TCA health providers, were examined. We also carried out direct observations of healthcare delivery facilities to map the workings of the health system in each of the study states. In-depth interviews were conducted with a range of stakeholders, including policy elites, health administrators, TCA health providers and allopathic (modern medicine) providers appointed in government health facilities, local traditional healers, community health workers, village elders, opinion leaders, and representatives of community organizations. Interviews explored the stakeholders’ experiences and perceptions related to their roles, job responsibilities, and interactions with the various players in the health system. While 73 in-depth interviews were conducted in Kerala and 46 in Meghalaya, data collection is ongoing in Delhi. Further data collection is also planned in Kerala and Meghalaya.

## Results

Major hurdles in mainstreaming of TCA health providers in essential health service delivery include absence of, or limited, formal communication and coordination between actors representing different systems of medicine; diverse levels of collegiality, ranging from hostility to harmony; and dissonance between the expectations placed on TC.

A health providers and the amenities provided to them. Further observations included contestations by different strata of actors on validity and reliability of medical evidence from alternative systems; lack of coherence in the range of medical evidence across different systems of medicine; and weak conformance by state to the national policy in certain states, and limited action in communicating policy directives across the systems of medicines. Conflicting loyalties, to systems of medicine, patients, and the public health system, emerged as a key finding in the experiences and perceptions of TCA health providers in one state. Local health traditions implemented through family-based practice, although acknowledged as resonating deeply with the local culture, were found to have little, if any, place in the official health system. These gaps are compounded by the limited opportunities for formal collaborative work across different systems of medicine.

## Discussion

Preliminary findings suggest that on the one hand, national policy articulations are not adequately translated into the state context. On the other hand, these articulations leave unresolved larger ethical questions of validity and reliability of evidence, and inter-system coherence that could more concretely support integration efforts. There is a profound need to understand and amend state-specific health governance processes and institutional capacities, and engender receptivity in mainstream health systems to alternative approaches, for the evolution of a truly integrated workforce for health services in India and other low- and middle-income countries. This may require institutionalizing incentives and opportunities for routine interaction between practitioners across systems of medicine in training, and ideally, in practice.

## Competing interests

Members of the research team for this study included allopathic (modern medicine) and homoeopathic doctors.

## Funding statement

This study was funded by the Wellcome Trust Capacity Building Large Research Grant (No. F012/RG/KS/02).

